# Supplementary material for: Interaction of Oxicam Derivatives with the Artificial Models of Biological Membranes—Calorimetric and Fluorescence Spectroscopic Study
Source: Membranes (Basel). 2022 Aug 17;12(8):791. doi: 10.3390/membranes12080791 (PMC9412344; doi:10.3390/membranes12080791)
Supplement: Supplementary file 1 [file membranes-12-00791-s001.zip › membranes-1863424-supplementary.pdf]

Supplementary Materials

Interaction of Oxicam Derivatives with the Artificial Models of Biological Membranes—Calorimetric and Fluorescence Spectroscopic Study

Jadwiga Maniewska [1,*], Żaneta Czyżnikowska [2], Berenika M. Szczęśniak-Sięga [1], and Krystyna Michalak [3]

[1] Department of Medicinal Chemistry, Faculty of Pharmacy, Wroclaw Medical University, Borowska 211, 50-556 Wrocław, Poland
[2] Department of Inorganic Chemistry, Faculty of Pharmacy, Wroclaw Medical University, Borowska 211a, 50-556 Wrocław, Poland
[3] Department of Biophysics and Neuroscience, Faculty of Medicine, Wroclaw Medical University, Chałubińskiego Poland

* Correspondence: jadwiga.maniewska@umw.edu.pl; Tel.: +48-71-784-03-97

Table S1. Absorption and distribution of studied oxicam derivatives.

Parameter	Compound					
	PR1	PR2	PR12	PR26	PR27	PR38
HIA (Human Intestinal Absorption; Category 1: HIA+(HIA < 30%); Category 0: HIA-(HIA < 30%); The output value is the probability of being HIA+)	0.3	0.04	0.01	0.02	0.01	0.01
Caco-2 Permeability; optimal higher than -5.15 log cm/s	-5.7	-5.8	-5.7	-5.5	-5.4	-5.7
MDCK Permeability; high passive permeability: $>20\cdot10^{-6}$	$3.2\cdot10^{-5}$	$2.9\cdot10^{-5}$	$2.9\cdot10^{-5}$	$3.4\cdot10^{-5}$	$3\cdot10^{-5}$	$3.1\cdot10^{-5}$
F 20% (20% Bioavailability; Category 1: F20%+ (bioavailability < 20%); Category 0: F20%- (bioavailability ≥ 20%); The output value is the probability of being F20% +)	0.5	0.4	0.1	0.01	0.03	0.01

F 30% (30% Bioavailability; Category 1: F30%+ (bioavailability < 30%); Category 0: F30%- (bioavailability ≥ 30%); The output value is the probability of being F30% +)	0.9	0.9	0.01	0.03	0.04	0.1
PPB (Plasma Protein Binding; Optimal < 90%. Drugs with high protein-bound may have a low therapeutic index)	98%	98%	98%	98%	98%	98%
VD (Volume Distribution; Optimal 0.04-20L/kg)	1.9	0.4	0.4	1.9	0.4	1.8
BBB Penetration (Blood-Brain Barrier Penetration Category; 1: BBB+; Category 0: BBB-; The output value is the probability of being BBB+)	0.7	0.2	0.2	0.6	0.1	0.5

Table S2. Metabolism and excretion of oxicam derivatives.

Parameter	Compound					
	PR1	PR2	PR12	PR26	PR27	PR38
CYP1A2 inhibitor	no	no	no	no	no	no
CYP2C19 inhibitor	yes	yes	yes	yes	yes	yes
CYP2C9 inhibitor	yes	yes	yes	yes	yes	yes
CYP2D6 inhibitor	yes	no	no	no	no	yes
CYP3A4 inhibitor	yes	yes	yes	yes	yes	yes
CL Clearance (High: >15 mL/min/kg; moderate: 5-15 mL/min/kg; low)	4.2	3.4	4.0	4.2	3.1	5.3
T 1/2Category (1: long half-life ; Category 0: short half-life; long half-life: >3h; short half-life: <3h)	0.06	0.2	0.09	0.02	0.08	0.02

Table S3. Toxicity of oxicam derivatives.

Parameter	Compound					
	PR1	PR2	PR12	PR26	PR27	PR38
hERG Blockers, (heart rhythm disturbances) 1: active; 0: inactive	0.6	0.07	0.07	0.7	0.1	0.7
H-HT Human Hepatotoxicity 1: positive(+); 0: negative(-)	0.9	0.9	1.0	1.0	0.9	1.0
DILI Drug Induced Liver Injury 1: drugs with a high risk; 0: drugs with no risk.	1.0	1.0	1.0	1.0	1.0	1.0
AMES Toxicity 1: Ames positive(+); 0: Ames negative(-)	0.3	0.05	0.05	0.4	0.07	0.2
Rat Oral Acute Toxicity 0: low toxicity; 1: high toxicity	0.2	0.8	0.8	0.2	0.7	0.2
Skin Sensitization 1: Sensitizer; 0: Non-sensitizer	0.04	0.06	0.04	0.03	0.04	0.04
Carcinogen 1: carcinogens; Category 0: non-carcinogens	0.1	0.4	0.4	0.1	0.4	0.1
Eye Irritation 1: irritants; 0: nonirritants	0.007	0.03	0.006	0.006	0.006	0.006
Respiratory Toxicity 1: respiratory toxicants; 0: respiratory nontoxicants	0.2	0.1	0.08	0.1	0.075	0.1
